# The 22nd annual Nucleic Acids Research Web Server Issue 2024

**DOI:** 10.1093/nar/gkae492

**Published:** 2024-06-18

**Authors:** Dominik Seelow

**Affiliations:** Exploratory Diagnostic Sciences, Berlin Institute of Health at Charité – Universitätsmedizin Berlin, 10117Berlin, Germany; Institute of Medical Genetics and Human Genetics, Charité – Universitätsmedizin Berlin, corporate member of Freie Universität Berlin and Humboldt-Universität zu Berlin, 13353Berlin, Germany

This is the 22th edition of Nucleic Acids Research's Web Server Issue. This year, we feature 74 web servers offering solutions for ‘common’ problems (e.g. protein structure or variant effect assessment) but also for rather uncommon topics such as paleogenomics.

Publishing in the Web Server Issue is a multi-staged process: Authors must upload a short proposal about a functional web server by 20 December (submitting earlier is greatly encouraged and will usually lead to a faster decision). In a first step, I will evaluate proposal and web server for their general suitability. In case of a positive outcome, the web server will undergo in-depth testing by our internal ‘technical reviewers’. They (and I) also provide suggestions for improving the website. Should we consider an application good enough for publication, the authors are invited to submit a manuscript which will then undergo the normal peer-review process.

This year, we received 248 proposals and invited 83 (1/3) to submit a manuscript. I had a (potential) conflict of interest with five proposals. The whole process for these was handled by my fellow editors Janusz Bujnicki and Dan Rigden. Technical reviewers with a conflict of interest were of course not included.

This pre-selection process paid off: 89% of the manuscripts were accepted after peer-review. Whilst it might be frustrating for authors not to be allowed to submit a manuscript, it drastically reduces the burden on the reviewers. I know from my own experience how frustrating it is to review a web application that does not really work. Our referees are not beta testers and they are doing their reviews on a voluntary basis. And we all need them to keep the peer-review system running. For this Issue, I asked 641 potential referees for reviews. Thank you to all who reviewed a manuscript or suggested alternative referees!

Another thank you goes of course to our internal ‘technical reviewers’. Our team has grown: Whilst Lennard Ostendorf left due to time constraints, Andrea Cappannini, Sunandan Mukherjee, Dominik Sordyl, Krzysztof Sulik (all from Warsaw), Shusruto Rishik (from Saarbrücken) and Maja Noack (from my group in Berlin) joined the team. This accelerated the technical review process and by 20 February > 95% of the initial decisions were sent. I apologize to everyone who had to wait longer.

My last round of acknowledgements goes to the team at NAR, especially to Martine Bernardes Silva (without whom I would have missed all deadlines), Rhiannon Meaden, Ajit Minj and the typesetters.

But now it's time to let the people behind the scenes, our technical reviewers, come to word:

**Andrea Cappannini**

*Międzynarodowy Instytut Biologii Molekularnej i Komórkowej w Warszawie, Warsaw, Poland*


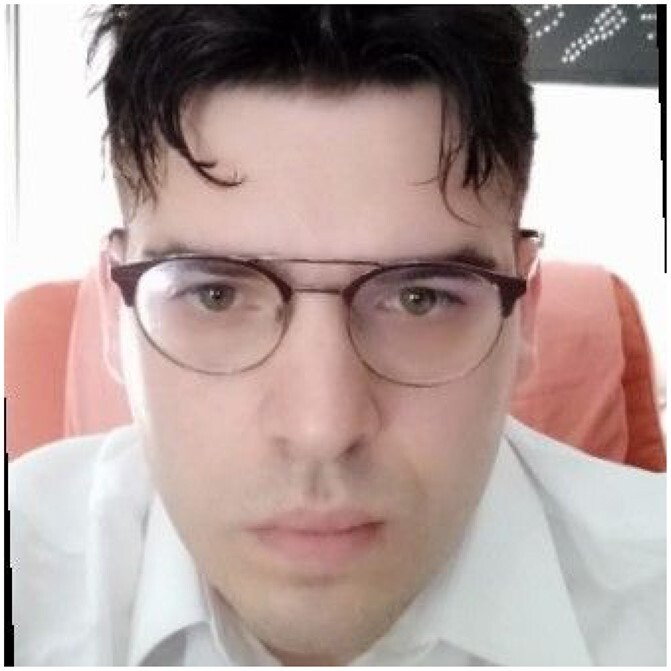


Participating as a reviewer for NAR allowed me to utilize the scholarly knowledge gained during my doctorate and years of collaboration with other scientific groups. This role facilitated a re-examination of certain biological principles that I had not recently considered. I am profoundly grateful for this opportunity.

**Oliver Küchler**

*Berliner Institut für Gesundheitsforschung in der Charité – Universitätsmedizin Berlin & Charité – Universitätsmedizin Berlin, Berlin, Germany*


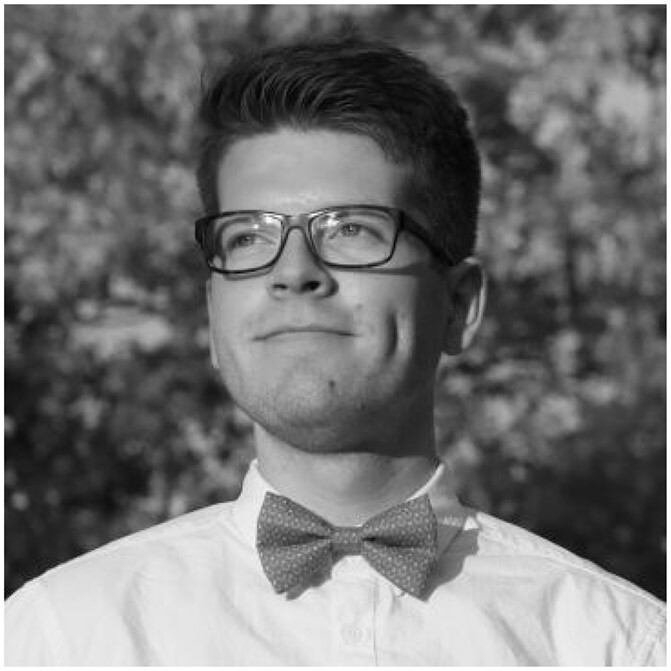


As an in-depth tester for the NAR Web Server Issue, I find it very exciting to closely engage with cutting-edge bioinformatics tools.

Evaluating their performances allows me to provide valuable feedback, contributing to the continuous improvement of scientific tools.

**Sunandan Mukherjee**

*Międzynarodowy Instytut Biologii Molekularnej i Komórkowej w Warszawie, Warsaw, Poland*


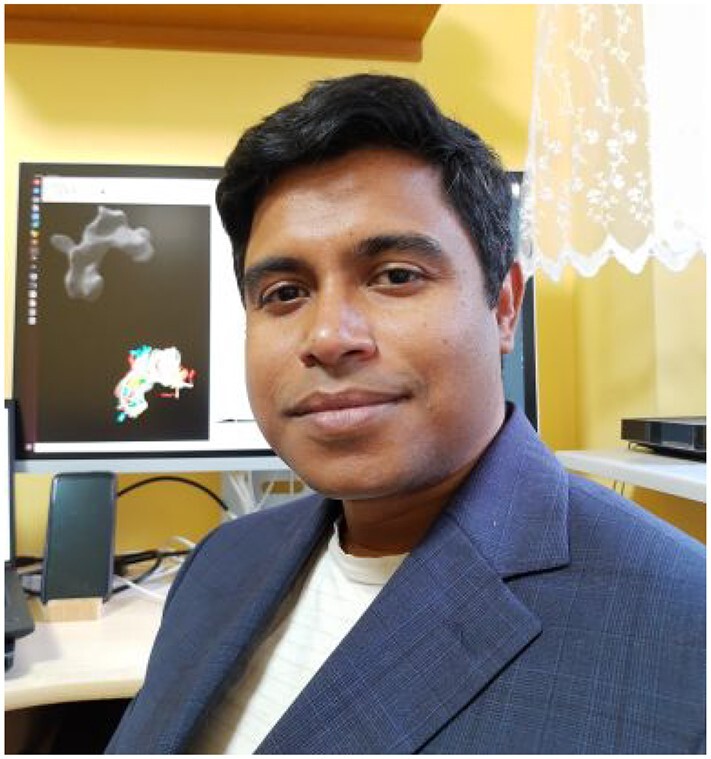


Serving as an in-depth tester for the NAR Web Server Issue afforded me valuable insights into the latest trends and advancements in bioinformatics. This role allowed me to thoroughly assess good (and bad) practices, which will be extremely helpful for me in the future. Overall, it was an enriching experience that provided me with a unique perspective on the editorial process.

**Maja Noack**

*Berliner Institut für Gesundheitsforschung in der Charité – Universitätsmedizin Berlin, Berlin, Germany*


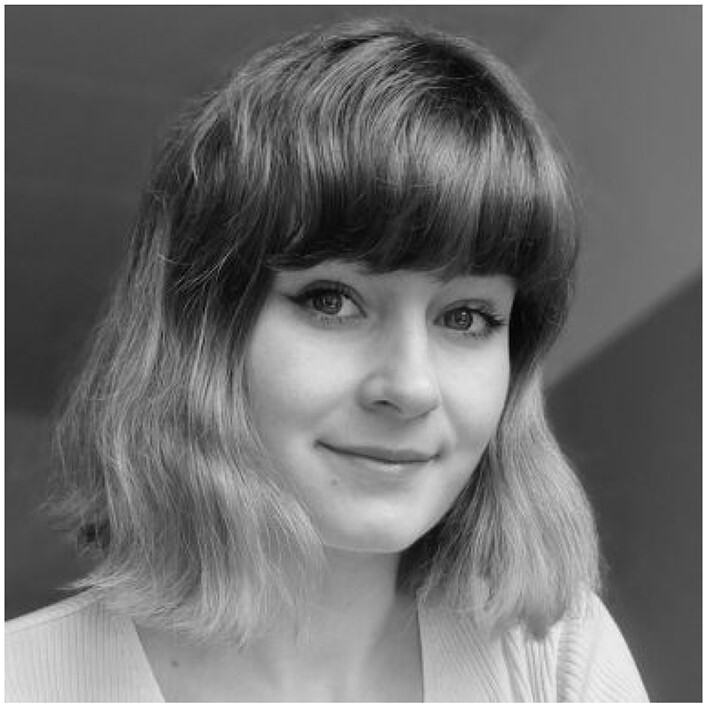


The opportunity to be a reviewer for the NAR Web Server Issue allows me to stay in touch with the latest developments in my field, evaluate new tools and provide constructive feedback to improve their functionality and performance. I enjoy the testing process particularly as it grants practical insights into exciting advancements and innovations.

**Shusruto Rishik**

*Universität des Saarlandes, Saarbrücken, Germany*


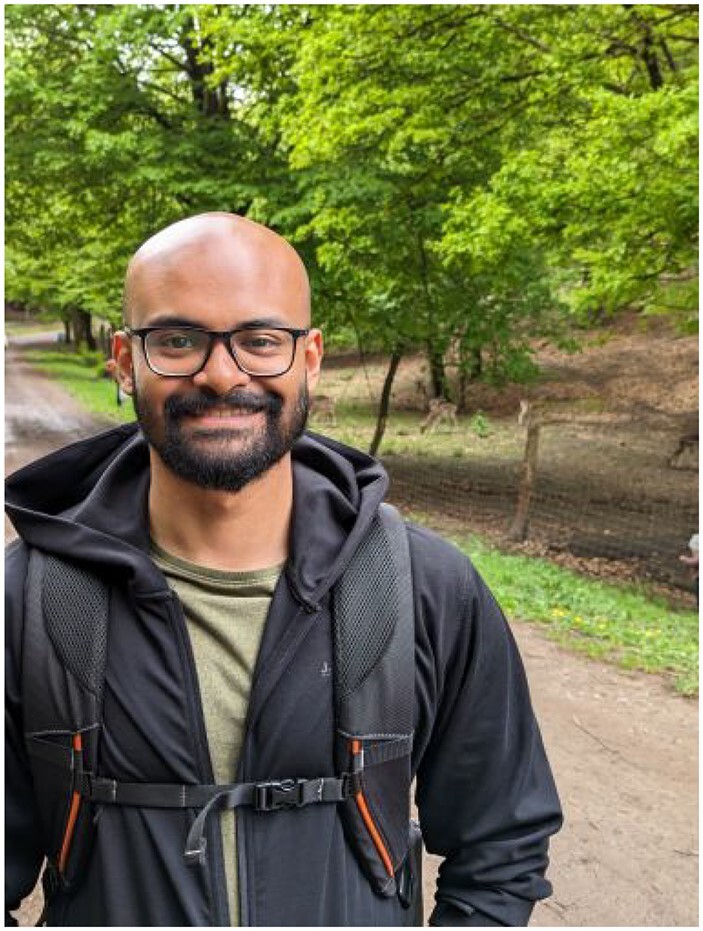


Science relies on volunteers to make itself run. I am passionate about the scientific method and wanted to contribute to helping keep it reliable.

**Georges Pierre Schmartz**

*Universität des Saarlandes, Saarbrücken, Germany*


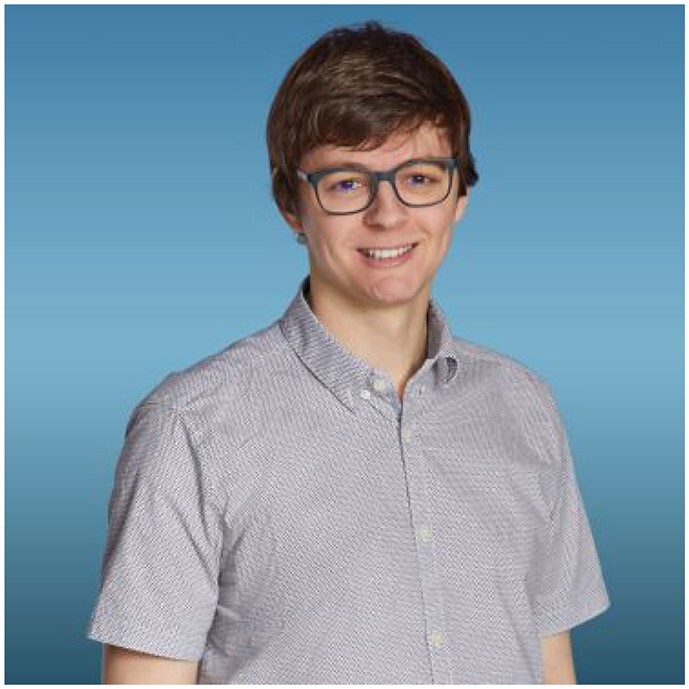


I am a PhD student at the Chair for Clinical Bioinformatics at Saarland University, specializing in the field of metagenomics. Recently, I transitioned into industry to work as a Data Scientist. Serving as an in-depth tester for *Nucleic Acid Research* enables me to stay abreast of the latest trends in my previous field of research and maintain connections within the scientific community.

**Adam Simpkin**

*University of Liverpool, Liverpool, UK*


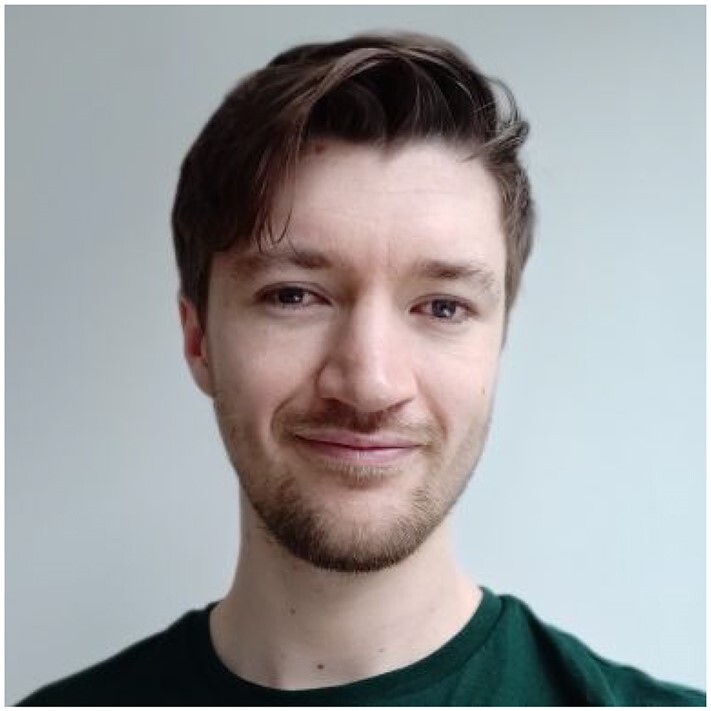


Working as an in-depth tester for *Nucleic Acids Research* allows me to stay up to date with new, interesting, and often unique bioinformatic tools. It is nice to be able to explore the functionality of these tools and provide meaningful feedback.

**Dominik Sordyl**

*Międzynarodowy Instytut Biologii Molekularnej i Komórkowej w Warszawie, Warsaw, Poland*


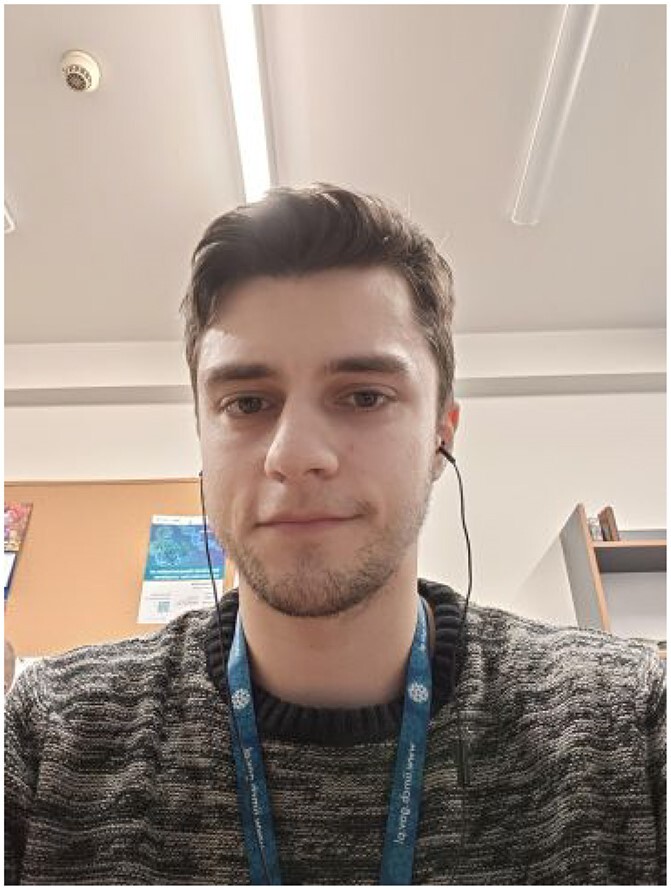


The most rewarding thing in being an in-depth tester is a feeling of contributing to the overall quality and reliability of web applications. As a person whose work focus kind of in between programming and doing science I found all of the new web servers interesting and informative. I also enjoy tackling new challenges, and testing some of the web servers was demanding.

**Krzysztof Sulik**

*Szkoła Główna Gospodarstwa Wiejskiego w Warszawie (SGGW), Warsaw, Poland*


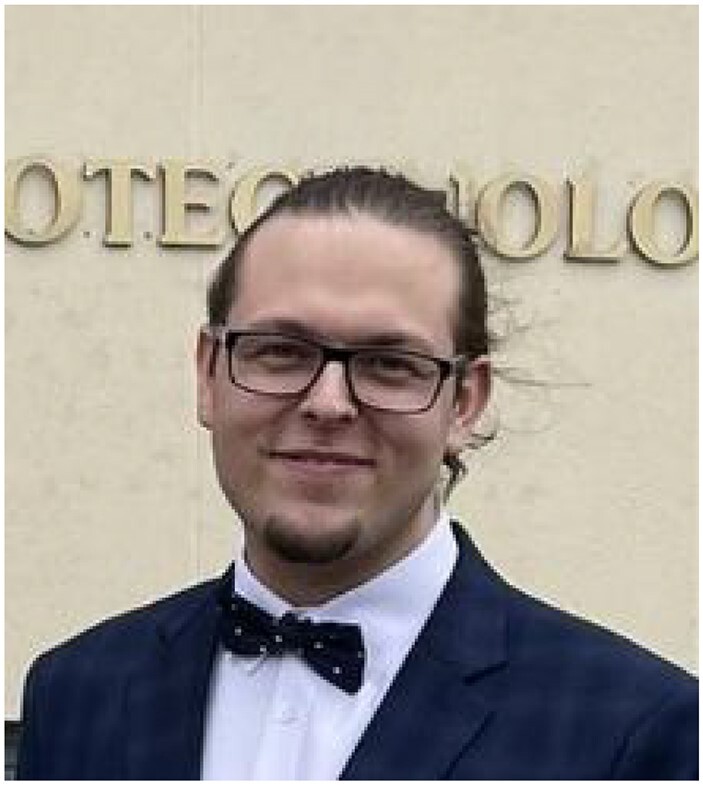


I really liked having the chance to evaluate the quality of the provided websites and being able experiment with their functionality.

**Viktoria Wagner**

*Universität des Saarlandes, Saarbrücken, Germany*


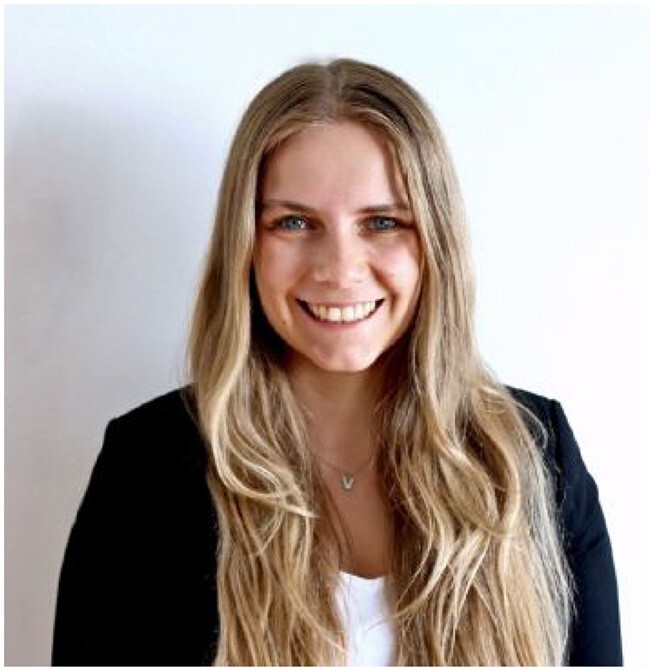


I like to do this job because it is really interesting to be part of an editorial team and learn more about the publishing process. Furthermore, it is exciting to see the great work of everyone submitting their work in NAR.

A very huge thank you to all of you! Your work made the 2024 Web Server Issue possible. It contains the following 74 manuscripts, ordered by their key area. As in the last years, nucleic acids play a smaller role than proteins, although the title of our journal suggests otherwise.

**Table:** 

Title	URL
**DNA**	
** Deep DNAshape webserver **: Prediction and real-time visualization of DNA shape considering extended k-mers	https://deepdnashape.usc.edu
** DNAforge **: a design tool for nucleic acid wireframe nanostructures	https://dnaforge.org/
** iM-Seeker **: a webserver for DNA i-motifs prediction and scoring via automated machine learning	https://im-seeker.org
**Evolution / genetics / genomics / genome editing**	
** AlPaCas **: Allele-specific CRISPR Gene Editing through a Protospacer-Adjacent-Motif (PAM) approach	https://schubert.bio.uniroma1.it/alpacas
Annotation and visualization of parasite, fungi and arthropod genomes with **Companion**	https://companion.ac.uk/
** ChIP-Atlas 3.0 **: A data-mining suite to explore chromosome architecture together with large-scale regulome data	https://chip-atlas.org/
** DORA **: An interactive map for the visualization and analysis of ancient human DNA and associated data	https://dora.modelrxiv.org/
Drawing human pedigree charts with **DrawPed**	https://www.genecascade.org/DrawPed/
** FEVER **: An interactive web-based resource for evolutionary transcriptomics across fishes	https://fever.sk8.inrae.fr/
** Imputation Server PGS **: An automated approach to calculate polygenic risk scores on imputation servers	https://imputationserver.sph.umich.edu
Interactive Tree Of Life (**iTOL**) v6: Recent updates to the phylogenetic tree display and annotation tool	https://itol.embl.de
The **Galaxy:** Platform for accessible, reproducible, and collaborative data analyses: 2024 update	https://galaxyproject.org
** LncRNAway **: A web-based sgRNA design tool for precise and effective suppression of long noncoding RNAs	https://www.lncrnaway.com
** mtDNA-Server 2 **: Advancing mitochondrial DNA analysis through highly parallelized data processing and interactive analytics	https://mitoverse.i-med.ac.at
** NeMu **: A comprehensive pipeline for accurate reconstruction of neutral mutation spectra from evolutionary data	https://nemu-pipeline.com/
** SeqCAT **: Sequence conversion and analysis toolbox	https://mtb.bioinf.med.uni-goettingen.de/SeqCAT/
** SynDesign **: Web-based prime editing guide RNA design and evaluation tool for saturation genome editing	https://deepcrispr.info/SynDesign
** TEENA **: An integrated web server for transposable element enrichment analysis in various model and non-model organisms	https://sun-lab.yzu.edu.cn/TEENA/
**Variant assessment**	
** MutationExplorer **: A webserver for mutation of proteins and 3D visualization of energetic impacts	http://proteinformatics.org/mutation_explorer/
** ProtVar **: Mapping and contextualising human missense variation	https://www.ebi.ac.uk/protvar
** REEV **: Review, evaluate and explain variants	https://reev.bihealth.org/
**Proteins**	
** AggreProt **: A web server for predicting and engineering aggregation prone regions in proteins	https://loschmidt.chemi.muni.cz/aggreprot/
** Aggrescan4D **: Structure-informed analysis of pH-dependent protein aggregation	https://biocomp.chem.uw.edu.pl/a4d/
** AIUPred **: Combining energy estimation with deep learning for the enhanced prediction of protein disorder	https://aiupred.elte.hu
** AlphaFind **: Discover structure similarity across the proteome in AlphaFold DB	https://alphafind.fi.muni.cz
** AlphaKnot 2.0 **: A web server for the visualization of proteins’ knotting and a database of knotted AlphaFold-predicted models	https://alphaknot.cent.uw.edu.pl/
** CASTpFold **: Computed Atlas of Surface Topography of the universe of protein Folds	https://cfold.bme.uic.edu/castpfold/
The **Damietta Server**: A comprehensive protein design toolkit	https://damietta.de/
** DDMut-PPI **: Predicting effects of mutations on protein–protein interactions using graph-based deep learning	https://biosig.lab.uq.edu.au/ddmut_ppi
** DeepLoc 2.1 **: Multi-label membrane protein type prediction using protein language models	https://services.healthtech.dtu.dk/services/DeepLoc-2.1/
** DEGRONOPEDIA **: A web server for proteome-wide inspection of degrons	https://degronopedia.com/
** Frustraevo **: A web server to localize and quantify the conservation of local energetic frustration in protein families	https://frustraevo.qb.fcen.uba.ar/
** GPS-SUMO 2.0 **: An updated online service for the prediction of SUMOylation sites and SUMO-interacting motifs	https://sumo.biocuckoo.cn/
** GPSFun **: Geometry-aware protein sequence function predictions with language models	https://bio-web1.nscc-gz.cn/app/GPSFun
** MinActionPath2 **: Path generation between different conformations of large macromolecular assemblies by action minimization	http://dynstr.pasteur.fr/servers/minactionpath/minactionpath2_submission
** PPI3D **: A web server for searching, analyzing and modeling protein–protein, protein–peptide and protein–nucleic acid interactions	https://bioinformatics.lt/ppi3d
** PROSCA **: An online platform for humanized scaffold mining facilitating rational protein engineering	https://idrblab.org/prosca/
** Proscan **: A structure-based proline design web server	https://proscan.ibbr.umd.edu
Deep learning for the **PSIPRED** Protein Analysis Workbench	http://bioinf.cs.ucl.ac.uk/psipred/
** PypKa Server **: Online p*K*_a_ predictions and biomolecular structure preparation with precomputed data from PDB and AlphaFold DB	https://pypka.org
** REME **: An integrated platform for reaction enzyme mining and evaluation	https://reme.biodesign.ac.cn/
** RING 4.0 **: Faster residue interaction networks with novel interaction types across over 35 000 different chemical structures	https://ring.biocomputingup.it/
** SLiMAn 2.0 **: Meaningful navigation through peptide-protein interaction networks.	https://sliman2.cbs.cnrs.fr
The **structure assessment web server**: for proteins, complexes and more	https://swissmodel.expasy.org/assess
** SwissDock 2024 **: major enhancements for small-molecule docking with attracting cavities and AutoDock Vina	https://dev.swissdock.ch/
** WeSA **: A web server for improving affinity proteomics data	http://wesa.russelllab.org/
**RNA**	
** IsoVis **: A webserver for visualization and annotation of alternative RNA isoforms	https://isomix.org/isovis/
** RNAhugs **: Web server for customized 3D RNA structure alignment	https://rnahugs.cs.put.poznan.pl/
** RNAscape **: Geometric mapping and customizable visualization of RNA structure	https://rnascape.usc.edu/
** SHAPEwarp-web **: Sequence-agnostic search for structurally homologous RNA regions across databases of chemical probing data	https://shapewarp.incarnatolab.com
** SimRNAweb v2.0 **: A web server for RNA folding simulations and 3D structure modeling, with optional restraints and enhanced analysis of folding trajectories	https://genesilico.pl/SimRNAweb
** SingmiR **: A single-cell miRNA alignment and analysis tool	https://ccb-compute.cs.uni-saarland.de/singmir
**Metabolomics / lipidomics / microbiome**	
** GCMS-ID **: A webserver for identifying compounds from gas chromatography mass spectrometry experiments	https://gcms-id.ca/
** LipidSig 2.0 **: Integrating lipid characteristic insights into advanced lipidomics data analysis	https://lipidsig.bioinfomics.tw/
** MetaboAnalyst 6.0 **: Towards a unified platform for metabolomics data processing, analysis and interpretation	https://www.metaboanalyst.ca
** Mibianto **: Ultra-efficient online microbiome analysis through *k*-mer based metagenomics	https://www.ccb.uni-saarland.de/mibianto
** WebGestalt 2024 **: Faster gene set analysis and new support for metabolomics and multi-omics	https://2024.webgestalt.org
**Cheminformatics / pharmacokinetics**	
** ADMETLAB 3.0 **: An updated comprehensive online ADMET prediction platform enhanced with broader coverage, improved performance, API functionality and decision support	https://admetlab3.scbdd.com
** admetSAR3.0 **: A comprehensive platform for exploration, prediction and optimization of chemical ADMET properties	http://lmmd.ecust.edu.cn/admetsar3/
** ChemFH **: An integrated tool for screening frequent false positives in chemical biology and drug discovery	https://chemfh.scbdd.com/
** ChemFREE **: A one-stop comprehensive platform for ecological and environmental risk evaluation of chemicals in One Health world	http://chemfree.agroda.cn/chemfree/
** ChemoDOTS **: A web server to design chemistry-driven focused libraries	https://chemodots.marseille.inserm.fr/
** Deep-PK **: Deep learning for small molecule pharmacokinetic and toxicity prediction	https://biosig.lab.uq.edu.au/deeppk/
** DetSpace **: A web server for engineering detectable pathways for bio-based chemical production	https://detspace.carbonelllab.org/
** Drugst.One **: A plug-and-play solution for online systems medicine and network-based drug repurposing	https://drugst.one
** KinomeMETA **: A web platform for kinome-wide polypharmacology profiling with meta-learning	https://kinomemeta.alphama.com.cn/
** MolModa **: Accessible and secure molecular docking in a web browser	https://durrantlab.com/molmoda
** Pred-O3 **: A web server to predict molecules, olfactory receptors and odor relationships	https://odor.rpbs.univ-paris-diderot.fr/
** ProTox-3.0 **: A webserver for the prediction of toxicity of chemicals	https://tox.charite.de/
**Others**	
The **EMBL-EBI Job Dispatcher** sequence analysis tools framework in 2024	https://www.ebi.ac.uk/jdispatcher
Next-Generation **IEDB Tools**: A platform for epitope prediction and analysis	https://nextgen-tools.iedb.org
** SEMA 2.0 **: Web-platform for B-cell conformational epitopes prediction using artificial intelligence	https://sema.airi.net
** PubTator 3.0 **: An AI-powered literature resource for unlocking biomedical knowledge	https://www.ncbi.nlm.nih.gov/research/pubtator3/
** TTSBBC **: Triplex target site biomarkers and barcodes in cancer	https://kowalski-labapps.dellmed.utexas.edu/TTSBBC/

I hope you enjoy this collection of web servers and that you will find applications that can help you address your research questions!

Should you wish to submit a manuscript to the 2025 Web Server Issue, please check https://academic.oup.com/nar/pages/submission_webserver for the changes in the procedure. The team at NAR and the scientific community are looking forward to novel and user-friendly web applications.

